# Cartilage Defect Treatment Using High-Density Autologous Chondrocyte Implantation (HD-ACI)

**DOI:** 10.3390/bioengineering10091083

**Published:** 2023-09-13

**Authors:** Pedro Guillén-García, Isabel Guillén-Vicente, Elena Rodríguez-Iñigo, Marta Guillén-Vicente, Tomás Fernando Fernández-Jaén, Ramón Navarro, Lucía Aboli, Raúl Torres, Steve Abelow, Juan Manuel López-Alcorocho

**Affiliations:** Department of Traumatology and Research Unit, Clínica CEMTRO, 28035 Madrid, Spaintomas.fernandez@clinicacemtro.com (T.F.F.-J.);

**Keywords:** cultured chondrocytes, autologous chondrocyte implantation, cell therapy, tissue engineering, high cell density

## Abstract

Hyaline cartilage’s inability to self-repair can lead to osteoarthritis and joint replacement. Various treatments, including cell therapy, have been developed for cartilage damage. Autologous chondrocyte implantation (ACI) is considered the best option for focal chondral lesions. In this article, we aimed to create a narrative review that highlights the evolution and enhancement of our chondrocyte implantation technique: High-Density-ACI (HD-ACI) Membrane-assisted Autologous Chondrocyte Implantation (MACI) improved ACI using a collagen membrane as a carrier. However, low cell density in MACI resulted in softer regenerated tissue. HD-ACI was developed to improve MACI, implanting 5 million chondrocytes per cm^2^, providing higher cell density. In animal models, HD-ACI formed hyaline-like cartilage, while other treatments led to fibrocartilage. HD-ACI was further evaluated in patients with knee or ankle defects and expanded to treat hip lesions and bilateral defects. HD-ACI offers a potential solution for cartilage defects, improving outcomes in regenerative medicine and cell therapy. HD-ACI, with its higher cell density, shows promise for treating chondral defects and advancing cartilage repair in regenerative medicine and cell therapy.

## 1. Introduction

Hyaline cartilage is a specialized connective tissue that covers the ends of the bones in joints [1]. Chondrocytes, the only cell type of hyaline cartilage, are immersed in a dense extracellular matrix that consists of water, collagen (type II collagen mainly), aggrecan, hyaluronic acid and other minor components [1,2]. Hyaline cartilage is an aneural tissue without either blood or lymphatic vessels [3]. Its composition and features confer on hyaline cartilage its special properties: it is responsible for the low friction between bone surfaces in the joint and it is also able to provide shock absorption during movement [3,4]. Hyaline cartilage is unable to self-repair and symptomatic lesions, if left untreated, may progress to osteoarthritis, which may end in a total joint replacement [4]

Several treatment options to treat cartilage lesions have been developed. Table 1 shows the features of the most common surgical treatments to repair cartilage lesions. The microfracture technique is a surgical procedure utilized for treating cartilage damage through standard arthroscopy portals and drill holes [5]. Typically, this technique is applied to patients with small- to medium-sized cartilage defects. During the procedure, a sharp instrument is used to create multiple small fractures in the underlying bone. The fractures cause bleeding and form a clot, which eventually leads to the formation of fibrocartilage repair tissue [5,6,7,8].

Osteochondral grafting is a surgical procedure that aims to transplant healthy cartilage and bone from one body region to an injured one [9]. Mosaicplasty and OATS (Osteochondral Autograft Transfer System) are two osteochondral grafting methods that involve transferring healthy bone and cartilage from one body area to a damaged region [10,11,12].

Osteochondral allograft (OCA) transplantation serves as a treatment solution for a range of joint conditions, including sizable osteochondral defects stemming from osteonecrosis, osteochondritis dissecans, and post-traumatic injuries [13]. This technique is progressively gaining popularity among young athletes who possess cartilage defects and are unsuitable candidates for knee arthroplasty. The distinct advantage of OCA transplantation, in comparison to other cartilage procedures such as arthroscopic debridement, microfracture, or osteochondral autograft transplantation, lies in its ability to effectively address lesions larger than 2 cm^2^. It involves a one-stage process that enables the transfer of size-matched allograft cartilage along with subchondral bone into knee articular defects. This stands in contrast to cell-based methods like autologous chondrocyte implantation, which often require repeated interventions to achieve desired outcomes [14,15].

Huge evidence concerning the use of regenerative medicine, especially cell therapy, has been published [16,17,18,19,20]. Cell therapy for cartilage repair is an innovative approach aimed at addressing cartilage damage and promoting tissue regeneration. This therapeutic technique involves the use of specialized cells, such as chondrocytes, mesenchymal stem cells (MSCs), or other progenitor cells, to stimulate cartilage growth and repair [21]. The goal of cell therapy is to replace or regenerate damaged cartilage, ultimately restoring joint function and alleviating pain in individuals with cartilage-related conditions [22]. While MSCs have shown potential in some preclinical studies and early-phase clinical trials, there are challenges and limitations to consider. Some studies have reported limited cartilage regeneration and integration of MSCs into the existing tissue. Moreover, the long-term outcomes of MSC-based therapies for cartilage repair remain uncertain. Additionally, the immunomodulatory and anti-inflammatory properties of MSCs may not always translate to consistent cartilage repair results, especially in more complex and severe cases [23].

Autologous chondrocyte implantation (ACI) is an advanced surgical procedure used for the repair of cartilage defects in joints, particularly in the knee. The technique involves the transplantation of a patient’s own healthy cartilage cells (chondrocytes) into the damaged area to stimulate cartilage regeneration and repair [24,25,26]. The goal of ACI is to promote the formation of new cartilage tissue and repair the damaged area, thereby improving joint function and reducing pain. ACI is often considered for individuals with larger cartilage defects or when other conservative treatments have not been effective. The second iteration of ACI has evolved by integrating biomaterials. In particular, porcine type I/III collagen membranes have been employed in a procedure called membrane-assisted chondrocyte implantation (MACI), as well as in our own approach, referred to as high-density autologous chondrocyte implantation (HD-ACI), emphasizing a high chondrocyte density. ACI techniques are two-step procedures, implying two surgeries: the first surgery to take a healthy cartilage biopsy from a non-bearing area, and the second one to implant the cultured chondrocytes [27].

This paper’s objective is to provide a narrative chronological overview of the progression towards conceptualizing the augmentation of cell-density. Its intention is to offer readers a comprehensive understanding of the idea’s emergence, the extensive experimentation it underwent, and the subsequent patient outcomes post-treatment. It starts with the genesis of the idea, followed by the proof-of-concept experiment conducted in an animal model. Subsequently, we discuss the studies on biopsy sources and the evaluation of cultured cell quality, specifically focusing on telomere length. Finally, we present the results obtained from treating patients with chondral defects in the knee, ankle, or hip using HD-ACI.

**Table 1:** Characteristics of different surgical techniques used for cartilage repair.

Technique	Type of Surgery	Size of Defects	Long-Term Results	Tissue Neoformed	Adverse Events	Cost	References
Microfractures	Minimally invasive	Small	Limited	Fibrocartilage	Risk of re-operation	Low	[5,6,7,8]
OATS/ Mosaicplasty	Invasive	Small to large	Limited	Hyaline cartilage	Related to donor site	Medium	[9,10,11,12]
Allograft	Invasive	Small to large	Limited	Hyaline cartilage	Allograft rejection	Medium	[13,14,15]
MSCs	Invasive	Small to large	Limited	Fibrocartilage	Risk of re-operation	High	[21,22,23]
ACI	Invasive	Small to large	Durable	Hyaline cartilage	Related to periosteum harvesting	High	[24,25]
MACI	Invasive	Small to large	Durable	Hyaline cartilage	Soft tissue with low cellularity	High	[26,27]

OATS, osteochondral autograft transfer system; MSCs: mesenchymal stem cells; ACI, autologous chondrocyte implantation; MACI, membrane-assisted chondrocyte implantation.

## 2. Rationale for HD-ACI: In Vitro and In Vivo Experiments

ACI in liquid medium was first used by our team in 1996, with 152 patients treated using this technique [28]. Considered as a whole, after 21–26 years of follow-up, results have been excellent, with 75% of patients returning to their normal life and even to practicing high-performance sports [28]. The use of ACI in liquid medium was extended until 2001, when we started using MACI, which was applied in the next 174 patients from 2001 to 2010 [29]. The results of the first 50 patients with 2 years of follow-up were published in 2015 [29]. Apart from the use of MACI as a first option to treat chondral defects, this technique was also used as a rescue from other approaches such as microfractures or mosaicplasty. The percentage of patients with good or excellent results was 89% in patients in whom MACI was used as a first treatment option and 69% in those who underwent other options to treat cartilage lesions before MACI.

**Figure 1:**
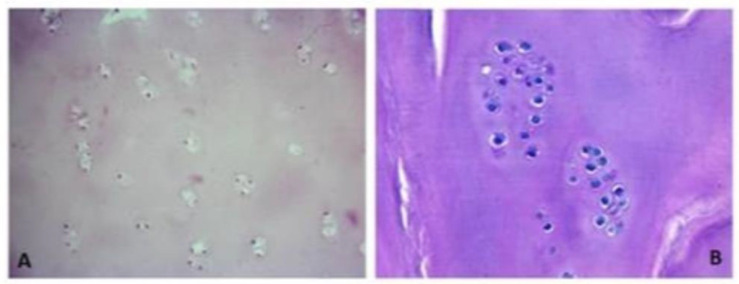
The histological slides stained with hematoxylin–eosin depict regenerated tissue biopsies obtained two years post membrane-assisted autologous chondrocyte implantation (MACI). The sections reveal limited chondrocytes, which are sparsely distributed either in clusters or in specific regions. These chondrocytes are found embedded within a matrix resembling hyaline cartilage. The images are captured at two different magnifications: (**A**) 200× magnification and (**B**) 400× magnification.

In some cases, we had to operate on some patients due to causes different to cartilage problems and when we reached the regenerated tissue, we noticed that it was softer than healthy tissue. In five cases, we could take a biopsy of the regenerated tissue two years after implantation (Figure 1). Histological analysis demonstrated the presence of scarce chondrocytes, distributed either in clusters or in columns in some areas and embedded in a hyaline matrix. We hypothesized that the reason could be that the cell density in MACI was much too low. In fact, in MACI, a total amount of 20 million cells was seeded in a 20 cm^2^ type I/III collagen membrane from porcine origin, so cell density was 1 million cells per cm^2^. For instance, if a lesion measured 3 × 2 cm, i.e., 6 cm^2^, a total amount of 6 million cells were implanted, with the remaining 14 million cells being wasted.

### 2.1. Experiments to Determine the Membrane Tolerance

We first investigated the maximum number of cells that the membrane used in MACI (Chondro-Gide, Geistlich Biomaterials, Wolhusen, Switzerland) could bear. To perform this experiment, cells were obtained from a biopsy of healthy cartilage taken in a non-bearing area of the knee, from a patient with a 3 × 3 cm chondral defect in the medial femoral condyle, who was going to undergo a MACI. A biopsy fragment was sent to Genzyme (Cambridge, MA, USA) for chondrocyte isolation and culture and the remaining fragment was sent to our laboratory. Chondrocytes were isolated from the remaining fragment, after digestion with 1 mg/mL collagenase A (Roche Diagnostics GmbH, Mannheim, Germany) at 37 °C overnight. The cells were cultured in complete medium (DMEM supplemented with 10% fetal bovine serum, L-Glutamine, and penicillin-streptomycin). Three passages were performed and a total number of 20 million cells was obtained.

Subsequently, the membranes were divided into ten segments, each measuring 1 cm^2^. These individual pieces were then placed within the wells of a six-well plate (Falcon^TM^, Fisher Scientific, Madrid, Spain) and seeded with varying quantities of chondrocytes, ranging from 1 million to 10 million cells. This resulted in a final cell density within the pieces ranging from 1 million cells per cm^2^ to 10 million cells per cm^2^. After waiting for 10 min, following the manufacturer’s recommended time for allowing membrane cell absorption, they were covered with 2 mL of complete medium and incubated for further 60 min. After time completion, supernatants were collected and centrifuged for 5 min at 500 g. Pellets were resuspended in 1 mL of DMEM.

**Figure 2:**
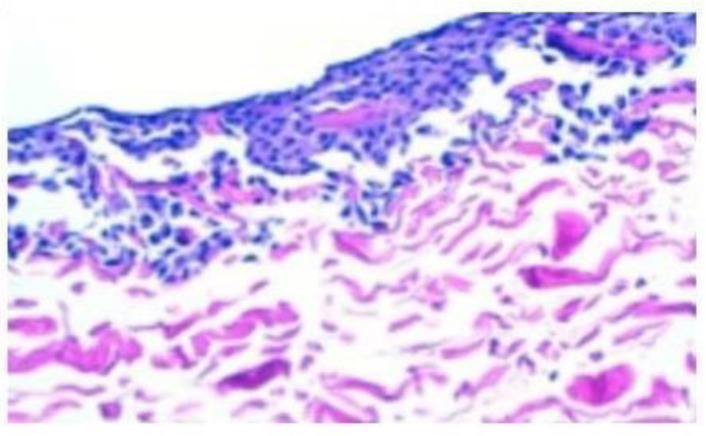
The hematoxylin–eosin staining provides confirmation of the existence of viable chondrocytes on the surface of all membrane pieces following incubation with varying amounts of chondrocytes, ranging from 1 million to 10 million cells.

To test the presence of cells in pellets and membranes, relative type II collagen and aggrecan expression was determined by RT-PCR. As shown in Table 2, expression of both genes could be confirmed in all membrane pieces; meanwhile, only the supernatants that came from 6–10 million chondrocytes expressed both type II collagen and aggrecan. These results suggested that when membranes were seeded with more than 6 million cells, they could not bear such a cell number and some of them escaped to the supernatant. Then, 10 µL of resuspended pellets were tested using the trypan-blue exclusion method in a Neubauer’s chamber for cell presence. Furthermore, hematoxylin–eosin staining was used to confirm chondrocyte presence in membrane pieces. These two methods allowed us to confirm both the presence of viable chondrocytes in supernatants coming from 6 to 10 million chondrocyte incubations and in all membrane pieces, with cells disposed at the membrane surface (Figure 2). Taking all these results together, we could conclude that porcine type I/II collagen membranes used in MACI could bear a maximum of 5 million chondrocytes/cm^2^ without losing any cell after incubation. For this reason, we established this cell density in our own cell implantation development, that we called high-density autologous chondrocyte implantation (HD-ACI).

### 2.2. HD-ACI Efficacy and Safety in the Sheep Animal Model

Our next step was to check HD-ACI efficacy and safety in a large animal model. We chose the sheep model and 5 million chondrocytes/cm^2^ (HD-ACI cell density) implants were compared with 1 million chondrocytes/cm^2^ (MACI cell density) and 1 million mesenchymal stem cells (MSC)/cm^2^ (Animal Committee Number: N-00660/2008) [30]. The study was performed in 2–3 years-old female sheep (Ovis aries, var. Manchega), divided into three groups according to the treatment that they are going to receive: Group 1: 1 million chondrocytes/cm^2^, Group 2: 5 million chondrocytes/cm^2^, and Group 3: 5 million MSC/cm^2^.

As the first step, animals were operated on in order to injure knee cartilage and to take the biopsies for cell isolation. Thus, in all animals, two 1 cm^2^ lesions in the hyaline cartilage of the medial femoral condyle and trochlea groove were performed. The first one was going to be treated with the correspondent cell implant according to the group and the removed cartilage was used as source tissue for chondrocyte culture. The second lesion, performed in the trochlea, was treated with microfractures. An adipose tissue biopsy from Hoffa’s fat pad was also taken in the animals from group 3, as a MSCs source.

Chondrocytes and MSCs were isolated from both cartilage and Hoffa fat pad biopsies and isolated cells were cultured for 22 days when 5 million cells were required for surgery (Groups 2 and 3), and 15 days in the case of 1 million cells (Group 1). When the appropriate cell quantity was reached, animals were re-operated to implant them into the medial femoral condyle cartilage lesions performed in the first surgery. Thus, cells were seeded onto a 1 cm^2^ piece of porcine I/III collagen membranes (Chondro-Gide; Geistlich Biomaterials) and after waiting 10 min, the membrane with the absorbed cells was attached to the lesion by suturing to the adjacent cartilage. Animals were euthanized 12 weeks after implantation and three types of samples were studied: biopsy from tissue generated at the medial femoral condyle after cell implantation, tissue generated at the trochlear groove after microfractures, and normal healthy hyaline cartilage taken in trochlear groove in an area close to that treated with microfractures. Tissue architecture and proteoglycan content was studied by hematoxylin–eosin and safranin-O fast green staining. Type I and II collagen as well as aggrecan expressions were tested by RT-PCR.

**Figure 3:**
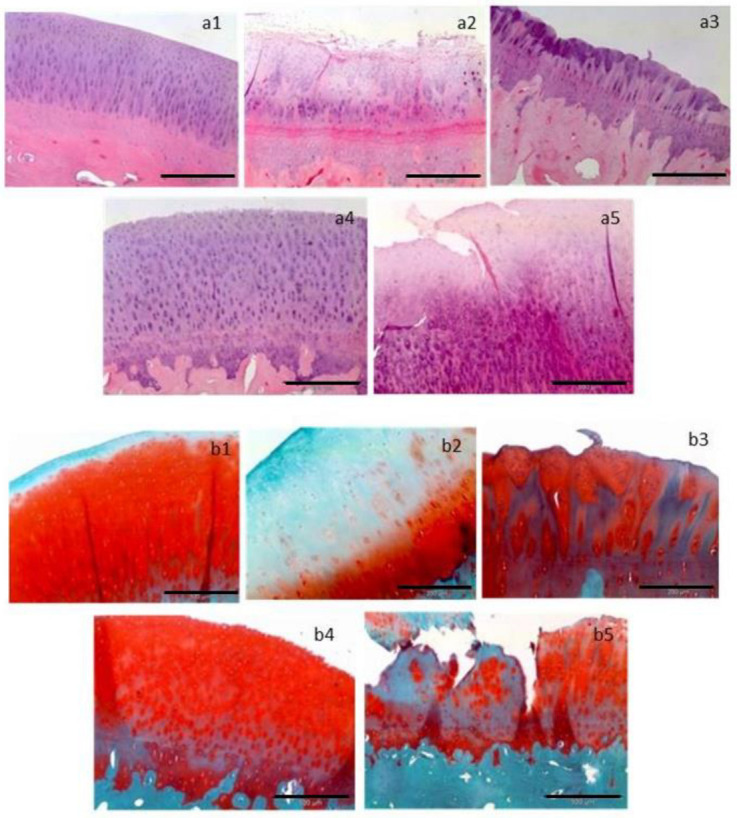
Representative histological sections are presented, stained with hematoxylin–eosin (**a1**–**a5**) to visualize tissue architecture, and with Safranin-O/Fast Green (**b1**–**b5**) to assess the mucopolysaccharide content of the cartilage matrix. The following treatments are depicted: (**a1**,**b1**) control, (**a2**,**b2**) microfracture, (**a3**,**b3**) implant seeded with 1 million chondrocytes per cm^2^, (**a4**,**b4**) implant seeded with 5 million chondrocytes per cm^2^, and (**a5**,**b5**) implant seeded with 5 million mesenchymal stem cells per cm^2^. Horizontal black bars represent a length of 200 µm [30].

Histological analysis of samples is shown in Figure 3. Hematoxylin–eosin staining of sample from microfractures (Figure 3(a2)) was quite different compared with the control ones (Figure 3(a1)), with elongated cells typical of fibrocartilage. In the case of samples obtained from chondrocyte implants (Figure 3(a3,a4)), a tissue that could be identified as hyaline-like cartilage was observed, with architecture and histological features very similar to the control in the case of 5 million chondrocyte implants (Figure 3(a4)). Finally, a fibrocartilage-like tissue was found in samples taken from the 5 million MSCs implants (Figure 3(a5)). Safranin-O fast green staining revealed that sections from animals implanted with a chondrocyte density of 5 million cells per cm^2^ (Figure 3(b4)) presented staining patterns quite close to those of control samples (Figure 3(b1)).

Expression of type II collagen and aggrecan, proteins that are normally expressed in the hyaline cartilage, was higher in control samples, followed by samples taken from 5 million and 1 million chondrocyte implants than in those taken from microfractures or 5 million MSCs. Exactly the opposite expression pattern was found in the case of type I collagen, which is mainly expressed in fibrocartilage. In this case, expression was higher in samples taken from 5 million MSCs implants and microfractures than in those from 1 million or 5 million chondrocytes and control.

The study detailed above, and its promising results, encouraged us to check HD-ACI treatment safety and efficacy in patients with chondral defects in knee or ankle. Later on, we even used this technique to treat chondral lesions in the hip or to treat patients with bilateral knee or ankle lesions.

### 2.3. Viability of Cultured Chondrocytes: Telomere Length Measurement

One of the primary concerns that arises during the implementation of cell therapy as a treatment for any disease is the viability and quality of cells that will be implanted into patients. In the case of tissues like hyaline cartilage, which are typically quiescent and have a low proliferation rate in their intact form, the cells are extracted from their natural environment and stimulated to divide under specific conditions. Cell culture is an artificial system that promotes cells to expand in vitro. In the case of cells for HD-ACI, chondrocytes are maintained until reaching enough functional cells to be implanted to patients, which usually takes us 4–6 weeks (three cell passages). Since cultured cells are subjected to stressing conditions in which they actively divide and telomere shortening is related to cell viability and even with the development of diseases such as osteoarthritis [31], we asked whether these culture conditions affect the telomere length. [32].

We studied cartilage biopsies from three patients with knee articular cartilage lesions that were going to undergo a HD-ACI. Biopsies were taken from a nonbearing area of the medial condyle and chondrocytes were isolated and cultured following the standard protocol established for HD-ACI. Briefly, cartilage biopsies suspended in Dulbecco’s Modified Eagle Medium (DMEM; Lonza Group Ltd., Basel, Switzerland) were processed in a sterile GMP (Good Manufacturing Practice) certified room, approved by the Spanish Health Authorities. Chondrocytes were isolated by digestion collagenase and cells were cultured in DMEM supplemented with 10% of autologous serum. When culture reached 80% confluence cells were detached and a maximum of three passages were performed until 40 to 50 million cells were obtained. After the third passage, a 1 million cells aliquot was reserved to be used for telomere length estimation, being the remaining cells implanted to patients. In each culture, the doubling time and the number of cells in each division were estimated by the trypan-blue exclusion method, assuming that cells grow exponentially. Telomere length was estimated by quantitative in situ fluorescent hybridization method (Q-FISH) in metaphasic cells [33].

**Figure 4:**
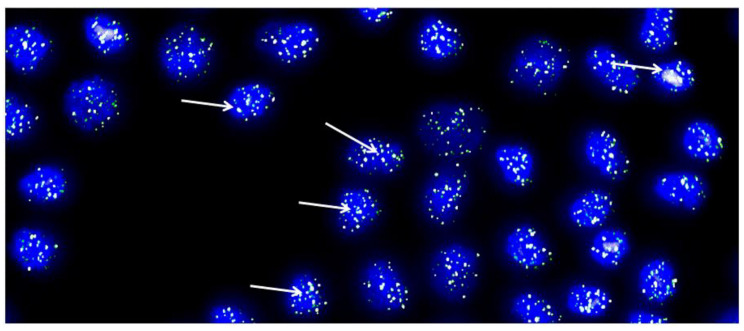
Representative metaphase image from chondrocytes cultured from each cartilage biopsy. The image showcases DAPI-stained nuclei in blue, while the telomeres are visualized as green/white fluorescent signals. The images were captured with the Leica Q-FISH software, using a linear acquisition mode and an integration time of 400 ms to avoid oversaturation of fluorescence intensity. The recordings were made with a COHU CCD camera on a Leica Leitz DMRB fluorescence microscope. Several arrows have been added to indicate the location of some telomeres as an example [32].

A representative example of Q-FISH in metaphasic cultured chondrocytes is depicted in Figure 4. Nuclei are stained in blue and telomeres in white-greenish fluorescence (indicated by arrows). After three passages, cells were in culture for a mean time of 45.67 days (±9.45 days), being the mean doubling time of 4.53 days (±0.71 days). It has been published that chondrocytes can become senescent after 30–35 population doublings, calling this cut-off Hayflick’s limit [34]. In our studied samples, cells divided a mean of 10.04 times (±0.82 times), which is three-folds smaller than Hayflick’s limit. On the other hand, regarding telomere measurement, in the three examined samples, the 20th percentile of the telomere length was 6.84, 6.96, and 7.06 kbp, respectively, and the median telomere span was 10.30, 10.47, and 10.73. These results mean that in 20% of the chondrocyte population studied, telomere length was 6.84–7.06 kbp while in 50% of them, telomere span was 10.30–10.73 kbp, which are long enough to think that most population of cultured cells are not senescent. These results allowed us to conclude that cultured chondrocytes for implantation are not senescent, at least in terms of telomere length and number of cell divisions involved in the procedure carried-out to obtain the necessary number of cells for HD-ACI. Although the number of studied samples was very little (only three), the low variation coefficients estimated from the telomere length measurement (3.54–3.94), made us affirm that our results are consistent enough to extrapolate to all chondrocyte cultures under our conditions.

### 2.4. Loose Bodies as a Source of Chondrocytes for Implantation

One of the first patients who fulfilled the inclusion criteria to receive a HD-ACI was a 18 year-old female whose diagnosis was osteochondritis dissecans (OCD) in the knee. During the arthroscopy performed to evaluate the chondral lesion and take a health cartilage biopsy, surgeons noticed that there was a loose body in the joint and they decided to send it to the laboratory together with the cartilage biopsy. Both samples were processed in a sterile room and only viable cells were obtained from the health cartilage, while cell culture could not be established from the loose body. After these results, we decided to start a study with loose bodies (PHC: pathologic hyaline cartilage) obtained from 34 patients (25 men and 9 women) of age 34.4 ± 11.7 years (Clinica CEMTRO Research Committee Number: CDI 008/13). [35]. In the study, inclusion criteria required patients to have a cartilage lesion in the knee that was diagnosed by MRI. However, patients with osteoarthritis grade III-IV Outerbridge or those whose cartilage damage was attributed to septic osteoarthritis or current tumoral disease were excluded from participation. The patients’ diagnosis was OCD in 11 cases, arthrosis in 13 patients, and trauma in the remaining 10 cases.

Based on the aspect of the PHC fragment, we classified the samples into three types: (a) fixed, when the PHC fragment was attached to the subchondral bone but beneath the fragment, the cartilage and the subchondral bone were not joined; (b) flapped fragment, when it was attached to the subchondral bone by one or more points and the fragment was almost loose; or (c) loose body when the PHC fragment was completely detached from the subchondral bone. In three cases (8.8%) the PHC fragment was fixed, in 6 patients (17.7%) the fragment was flapped, and in the remaining 25 (73.5%) the fragment was a loose body.

Half of each PHC sample and all health samples were processed to isolate and culture chondrocytes following the standard procedures. Expression of aggrecan (ACAN) and collagen types I and II (COL-I and COL-II) was studied in the remaining PHC sample by RT-PCR. In nine cases, we still had enough PHC tissue available to perform additional histological and immunohistochemical studies. In seven (20.6%) PHC fragments as well as in all the health cartilage samples taken at the same time as PHC samples, cell culture could be established. PHC samples in which the chondrocyte culture could be established had a significantly higher ACAN and COL-II expression than in the other samples in which the culture could not be set-up and which had a high expression of COL-I.

Our study demonstrated that not all the pathologic cartilage fragments detached from the knee articular cartilage are a good source of cells for a cell culture for a future autologous implantation, since chondrocyte culture could not be set-up in all of them. Our results suggest that the time elapsed between the loose body formation and the excision of the fragment is the main factor affecting cell culture setup, but there is another factor such as the degree of detachment between cartilage fragment and subchondral bone that must have to be taken into account.

## 3. Safety and Efficacy of HD-ACI in Patients with Chondral Lesions

### 3.1. Treatment of Chondral Lesions in the Knee

Our first study was performed in patients with chondral defects in the knee and its aim was to check short- and mid-term safety and efficacy of the HD-ACI procedure (Clinica CEMTRO Research Committee Number: CDI 014/09) [27]. In this study, patients were included if they met the following criteria: (i) outerbridge grade III-IV cartilage lesion in the knee (femoral condyles, trochlea, tibial plateau, or patella), diagnosed by an imaging test such as X-ray, magnetic resonance, or arthroresonance; (ii) had one to four lesions, each at least 1 cm^2^ in size; (iii) aged between 18 and 55 years. Patients were excluded from the study if they had any of the following conditions: (i) osteoarthritis; (ii) specular lesions (femoral condyle and tibial plateau from the same side); (iii) limb misalignment with more than 10° varus or valgus; (iv) meniscal lesions; (v) allergy to penicillin and/or streptomycin or hypersensitivity to bovine-derived products; (vi) active infection, tumoral pathology, or other systemic diseases such as rheumatoid arthritis or other autoimmune diseases with joint involvement.

The study included the first 50 patients (35 men and 15 women) who fulfilled the inclusion/exclusion criteria and had a follow-up period of at least 2 years. Patients’ median age was 35 years (18–49 years) and 22 of them had previous surgeries due to cartilage problems (microfractures, mosaicplasty, loose body extraction or different combinations among them). In these patients, a health cartilage biopsy (similar to three–four rice grains in size) from a non-bearing area (medial condyle) and processed in a sterile room certified by the Spanish Health Agency. Chondrocytes were isolated by enzymatic digestion and cultured to obtain 30–40 million cells and after 4–6 weeks, they were implanted in a second surgery. In the surgical act, chondral defects were debrided and measured to cut the porcine I/III collagen membrane (Chondro-Gide; Geistlich Biomaterials) according to their shape and size. Cells, resuspended in culture medium, were seeded onto the membrane, and then it was sutured to the adjacent bone and sealed with fibrin glue [27].

**Figure 5:**
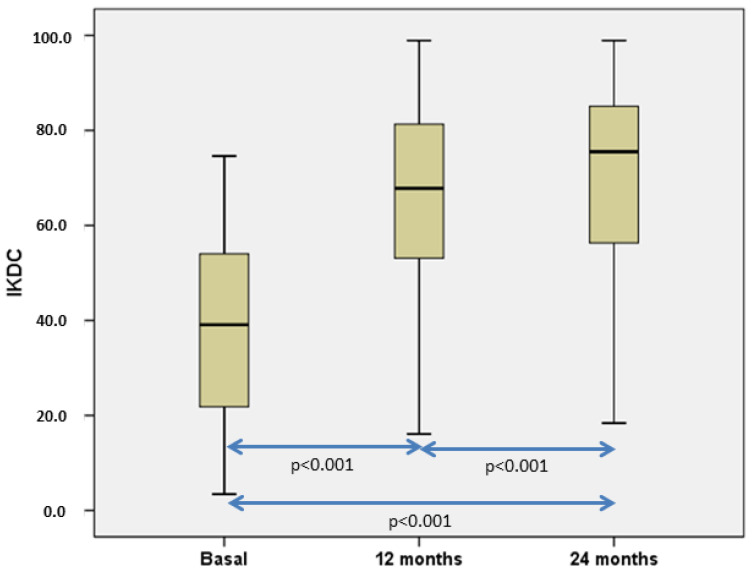
Boxplot representation illustrating the progression of International Knee Documentation Committee (IKDC) scores. Our analysis revealed statistically significant differences when comparing all IKDC scores collectively, and pairwise comparisons also reveal statistically significant results. [27].

The number of patients with pain significantly decreased from 50 (100%) patients before surgery to 17 (34%) and 11 (22%), 12, and 24 months, respectively, after chondrocyte implantation. On the other hand, the number of patients with swelling also significantly decreased from eight cases (16%) before cell implantation to two (4%) both at 12- and 24-months post-op. Patients’ subjective perception of knee symptoms and function before and 12 and 24 months after HD-ACI treatment was evaluated with the IKDC (International Knee Documentation Committee) questionnaire. Comparison of basal and 12 months and 24 months before surgery IKDC values showed a significant improvement (Figure 5). Mean IKDC score improvement in the 12-month follow-up visit was 26.3 points (95% CI = 18.2–34.4 points) and 31.0 points (95% CI = 22.9–39 points) at 24 months post-op. As the published Minimum Clinically Important Difference (MCID) for IKDC at 12 months is 16.7 points, IKDC improvement is perceived as a real amelioration.

The main limitations of this study were related to the lack of a second-look sample or an image to evaluate the novel tissue synthesized after HD-ACI, and it would also be desirable to include patients with more homogeneous clinical characteristics. However, our results’ robustness allowed us to conclude, at least from the clinical point of view, that HD-ACI is a safe and effective technique to treat cartilage defects in the knee.

### 3.2. HD-ACI for Chondral Ankle Lesions Treatment

In order to test HD-ACI safety and effectiveness in an additional joint, we performed a prospective study (Clinica CEMTRO Research Committee Number: CDI 002/10) in the first 24 patients (male/female: 14/10) with ankle cartilage injury who had a two-year follow-up and fulfilled the following criteria [36]. Inclusion criteria: International Cartilage Repair Society (ICRS) grade 3–4 cartilage lesion of the ankle (lateral or medial), diagnosed by imaging test (magnetic resonance or arthro-resonance), one to two lesions of at least 100 mm^2^ in size, and age ranging between 18 and 55 years. Exclusion criteria: arthrosis, misalignment of the limb (more than 10° varus or valgus), allergy to penicillin and/or streptomycin, hypersensitivity to bovine-derived products, active infection, tumoral pathology, and systemic diseases such as rheumatoid arthritis or other autoimmune diseases with articular involvement.

Median age (minimum–maximum) of patients was 31 years (18–55 years) and two of them had two lesions, so the number of operated lesions was twenty-six. Topographic chondral lesion location in the ankle was assessed following the Elias et al. [37] classification (three lesions in zone 1, two lesions in zone 3, sixteen lesions in zone 4, and five lesions in zone 6). Healthy cartilage biopsy was collected in each patient from a non-weight-bearing area (anterior talar neck) in a first arthroscopic surgery. Chondrocytes were isolated from cartilage biopsies and cultured until reaching a sufficient number of cells to perform HD-ACI. In the case of chondral lesions in zones 3 or 6 [37], HD-ACI was carried out by arthroscopy. When chondral lesions were located on zones 1 or 4, a medial malleolus osteotomy was performed. If a lesion was deeper than 4 mm, an adapted “sandwich” technique to fill the defect was performed [38]. Post-operative care included a mobilization program followed by progressive weight bearing with crutches and physical therapy sessions. At 4 months, stationary bicycle sessions and swimming were allowed, and at 9 to 10 months, they could jog slowly.

**Figure 6:**
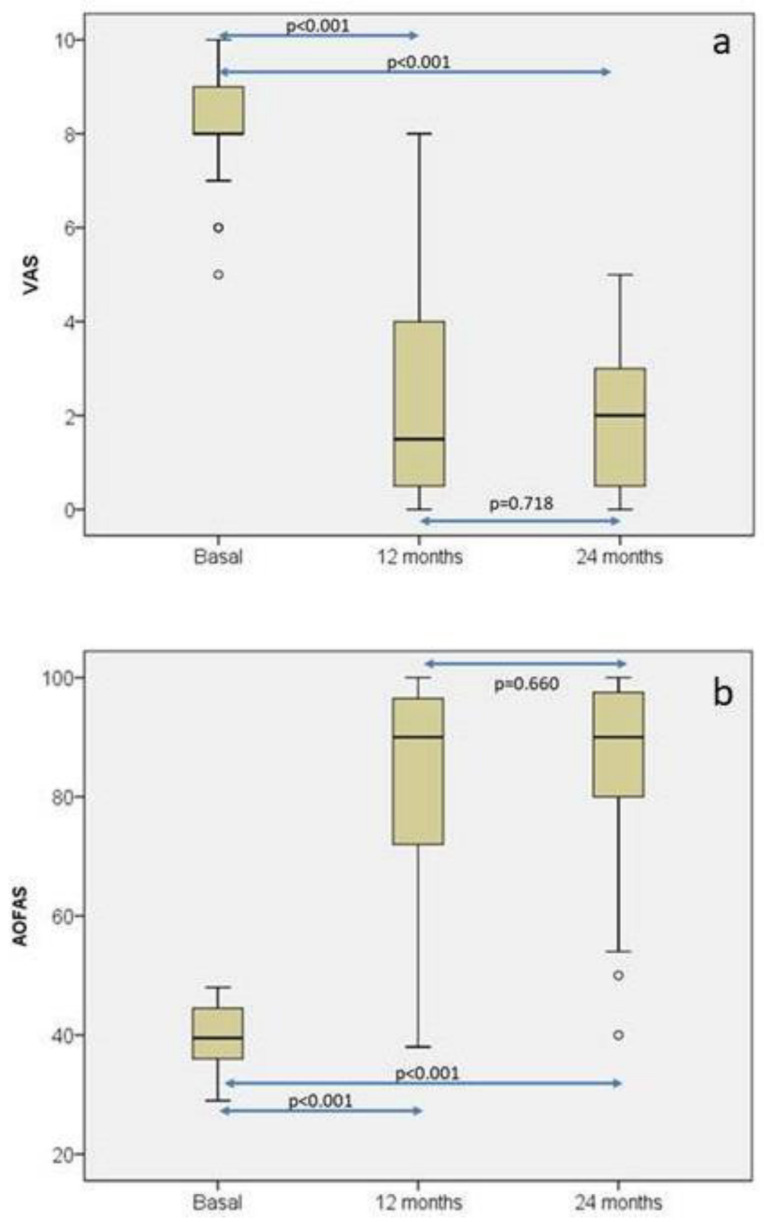
Box plot representations of the distributions of the visual analogue scale (VAS) (**a**) and the American Orthopedic Foot and Ankle Society (AOFAS) ankle-hindfoot score (**b**) at baseline, 12-month, and 24-month follow-up. Each symbol ◦ represents an outlier (data point that significantly deviates from the majority of other data points. The figure highlights statistically significant differences in both parameters across the different time points [36].

Twelve and twenty-four months after surgery, pain was evaluated with the Visual Analogic Scale (VAS) and ankle function with the American Orthopedic Foot and Ankle Society (AOFAS) ankle-hindfoot score [39]. As seen in Figure 6a, pain significantly decreased from baseline to 12 months. At that moment it reached a value that was maintained at 24 months. The behavior of AOFAS ankle-hindfoot score distribution was very similar to VAS (Figure 6b), so a significant increase was observed from baseline to 12 months (Mean AOFAS difference = 42.1 (95% CI of mean: 34.4–49.9)), with this difference also being maintained at 24-month follow-up (Mean AOFAS difference = 44.6 (95% CI of mean: 37.4–51.8)). In the case of AOFAS, to evaluate chondral defects in the ankle, MCID has not been established yet. However, Chan et al. [40] have determined that MCID for AOFAS ranges from 7.9 to 30.2 in hallux valgus surgery. In our patients, it was sufficiently higher than 30.2 to conclude that these differences in AOFAS score at 12 and 24 months with respect to baseline are perceived as a real improvement for the patients. Magnetic resonance observation of cartilage repair tissue (MOCART) [41] was used to assess cartilage healing at 12- and 24-month follow-up. Mean MOCART scores at 12- and 24-month follow-ups were 73.71 ± 15.99 and 72.33 ± 16.21, respectively. No factors such as gender, “sandwich” technique, laterality, location, number of lesions, lesion grade, or number of lesions had any influence on AOFAS or MOCART scores at 12 and 24 months.

As in the case of HD-ACI for chondral defects in the knee, the small number of patients included is the main limitation of this study. However, all publications on similar treatments such as ACI have similar case-series due to the low prevalence of talar cartilage lesions and, on the other hand, result robustness lead us to conclude that HD-ACI is a safe and effective technique to treat osteochondral lesions of the talus.

### 3.3. Treatment of Bilateral Chondral Defects with HD-ACI

The management of bilateral knee or ankle lesions presents a significant challenge, and there is a lack of scientific literature addressing this specific topic. Surgeons must make a decision regarding whether to perform separate procedures on each limb, one after the other, using different anesthesia, or to operate on both limbs simultaneously during a single surgical procedure using the same anesthesia. This decision should be based on the individual patient’s circumstances. Both procedures have advantages and disadvantages: If patients choose to undergo sequential surgeries, recovering from the first surgery before proceeding to the second, it is likely that they will require more time to regain their previous level of activity. This is because they will have to recover from two surgeries in total. However, on the positive side, during their recovery period, only one limb will be immobilized at a time, which may result in fewer mobility limitations. On the other hand, if both limbs are operated on simultaneously, patients will only need to recover from one surgery. However, they will face the inconvenience of having both limbs immobilized at the same time, which can pose challenges in terms of mobility during their recovery period.

In this sense, we examine the clinical outcome of eight patients with bilateral knee cartilage lesions [42] and two patients with bilateral ankle cartilage lesions [43] treated with HD-ACI on the same surgical act, followed 24 and 12 months, respectively (Clinica CEMTRO Research Committee Numbers: CDI 015/17 and CDI 002/18). In both cases, apart from the pain evaluated with the VAS and joint functionality assessed with the IKDC (knee) or AOFAS (ankle) questionnaires, we also evaluated the quality of life of patients with the EuroQol five-dimensional five-level questionnaire (EQ-5D-5 L). In the case of bilateral knee we included patients who fulfilled the following inclusion criteria: Outerbridge grade III-IV cartilage lesion in both knees diagnosed by an imaging test (X-ray, magnetic resonance, or arthro-resonance), one–four lesions of a minimum of 1 cm^2^ in size, and an age range from 16 to 55 years. The exclusion criteria: arthrosis, specular lesions (femoral condyle and tibial plateau from the same side), misalignment of the limb (more than 10° varus or valgus), meniscal lesions, allergy to penicillin and/or streptomycin, hypersensitivity to bovine-derived products, active infection, tumoral pathology, and systemic disease as rheumatoid arthritis or other autoimmune diseases with articular affectation. In the case of the ankle, we described the only two patients that we have with bilateral ankle cartilage lesions treated with HD-ACI.

**Figure 7:**
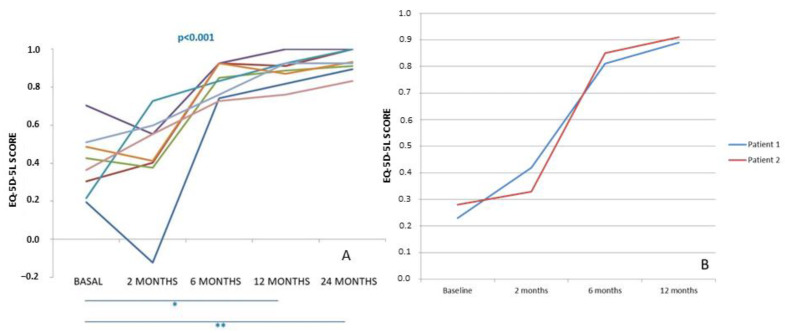
EQ-5D-5L score progression is observed in 8 patients with bilateral chondral lesions in the knees (**A**) and 2 patients in the ankle (**B**) who underwent treatment with high-density autologous chondrocyte implantation (HD-ACI). The scores are tracked from the baseline visit to the 24-month post-operative period Horizontal bars represent statistically significant pairwise comparison: basal vs. 12 months and basal vs. 24 months (* *p* < 0.05; ** *p* < 0.001). [42,43].

In both groups of patients, pain significantly decreased from very high values in the basal evaluation to normal or almost normal values at 12 months (ankle) or 24 months (knee) post-surgery. Subjective perception of joint function measured by the AOFAS or IKDC scores significantly increased at the end of follow-up, indicating that patients had an improvement in joint functionality with respect to baseline. Although pain and subjective perception are two parameters that could offer insights into the effectiveness of treatment, we deemed it crucial to consider the quality of life as a significant factor in assessing the overall impact of the treatment on patients. This consideration was particularly important as we administered treatment simultaneously to both limbs, with the goal of comprehensively evaluating its efficacy. In Figure 7, the evolution of EQ-5D-5L scores is depicted for the two distinct patient groups: eight patients who underwent knee surgery (A) and two patients who underwent ankle surgery (B) throughout the study period. The behavior of EQ-5D-5L was remarkably similar in both groups: it exhibited a decrease at 2 months post-surgery compared to the baseline value, followed by a gradual increase during the subsequent follow-up visits. Ultimately, the scores reached higher values at either 24 or 12 months after surgery than at the baseline assessment.

These studies represent an initial approach to simultaneously treating bilateral chondral defects in the knee or ankle using HD-ACI. The publications solely focus on describing the technique employed in a limited number of cases: eight patients with knee defects and two patients with ankle defects, with follow-up periods of 24 and 12 months, respectively. Despite the small sample size, the results obtained from both studies suggest that simultaneous treatment of bilateral chondral defects in the knee or ankle through a single surgical procedure with HD-ACI is a viable option. Patients who undergo this approach demonstrate satisfactory clinical outcomes and experience a positive impact on their quality of life.

### 3.4. HD-ACI for Chondral Defects in the Hip

Chondral defects affecting the articular cartilage in the hip can result in pain, limited mobility, and a reduced quality of life. The management of these defects requires a comprehensive strategy that incorporates non-surgical approaches as well as a range of surgical techniques. While individual cases may differ, the objective of these treatments is to alleviate pain, enhance joint function, and improve overall well-being. Among the surgical options available, regenerative medicine-based treatments offer a promising alternative worth considering. Consequently, we conducted an investigation into the advantages of HD-ACI treatment in two patients with localized chondral defects in the hip (femoroacetabular impingement; Clinica CEMTRO Research Committee Number: CDI 006/17). This is a case report of the two patients with hip chondral defects treated with HD-ACI in our institution. The patients were monitored for 36 months to evaluate the treatment’s long-term effectiveness [44].

There were no complications related to surgery reported by any of the patients. Prior to the operation, the patients had a pre-operative Visual Analog Scale (VAS) score of 4 and 2, respectively. However, both patients experienced complete resolution of pain at the 6-month mark following the surgery, and this pain relief was sustained throughout the entire follow-up period. To assess the quality of life, the International Hip Outcome Tool (iHOT-33) questionnaire was utilized, with baseline scores of 50 and 42 for the two patients. Remarkably, these scores significantly improved to 92 and 94 at the 36-month evaluation. Furthermore, the 3-year arthro-RMI results indicated that the cartilage defects were filled with a tissue similar to the surrounding healthy cartilage, further supporting the notion that HD-ACI is a reliable and effective technique for treating focal cartilage lesions in patients with advanced femoroacetabular impingement.

## 4. Discussion

In this paper, we aimed to review the process that led to the idea of increasing cell density in ACI, the proof-of-concept performed in an animal model, subsequent studies on biopsy sources and the quality of cultured cells in terms of telomere length, and the outcomes observed in patients with chondral defects in the knee or ankle who were treated with HD-ACI. When developing cell-based medicinal products like advanced therapy medicinal products (ATMPs), ensuring patient safety is of utmost importance. In the case of culturing chondrocytes for HD-ACI, we maintain stringent quality controls within a certified sterile environment following GMP. These controls encompass vital aspects of cell culture, including the preservation of cell viability, maintenance of cell morphology, ensuring purity, and upholding sterility. To assess the functionality of chondrocytes, we closely examine the expression of key markers such as aggrecan and type II collagen. Additionally, we limit the passage number to a maximum of three to mitigate the risk of cell dedifferentiation. Beyond these routine quality checks, we also emphasize the importance of genetic integrity tests for cell assessment. According to European regulatory authorities, conventional karyotyping is recognized as a valuable and effective technique for evaluating chromosomal stability during preclinical studies. In addition to karyotyping, other tests can be implemented for this purpose, including fluorescent in situ hybridization (FISH), array comparative genomic hybridization (aCGH), single nucleotide polymorphism (SNP) genotyping, and microsatellite instability (MSI) analysis. Notably, Wallenborn et al. [45] employed a combination of Giemsa staining banding, locus-specific FISH, spectral karyotyping, and SNP array techniques to test monolayer and spheroid chondrocyte cultures. Their study demonstrated that up to passage 3 in monolayer cell culture, no significant genetic instability occurred. However, prolonged cultivation times were associated with the emergence of polyploid metaphases and chromosomal aberrations. These findings, in conjunction with our telomere length analysis, provide evidence that monolayer chondrocyte cultures for HD-ACI are safe for application in patients.

Our results demonstrate the effectiveness and safety of HD-ACI in patients with focal chondral defects in the knee, ankle, and hip, as well as in patients with cartilage lesions in both knees or ankles. The findings have shown promising outcomes in terms of pain reduction and improvement in joint function across all treated joints. Furthermore, patients who underwent bilateral implantations reported an enhanced quality of life after undergoing a single procedure on both limbs. These results collectively indicate that HD-ACI is a reliable and effective technique for treating hyaline cartilage lesions.

The selection of different cartilage repair methods is contingent upon factors such as the extent and seriousness of the imperfection, patient characteristics, surgical proficiency, and available resources. Comparative research involving HD-ACI and other approaches remains absent in the current literature. Among our treated patients, some underwent HD-ACI treatment subsequent to the lack of success with alternative techniques like microfractures or MSC implantation [27,36]. Both microfractures and MSC implantation incite the formation of fibrocartilage, which is less enduring than native hyaline cartilage. Consequently, long-term outcomes can fluctuate since the repaired cartilage may not match the original cartilage in terms of quality [46,47,48]. Microfractures generally prove less effective for sizable or more severe cartilage defects, potentially affording only temporary relief in comparably minor cartilage imperfections [47].

In terms of mid-term outcomes, patients subjected to HD-ACI treatment for knee or ankle conditions demonstrate comparable results to those reported by other researchers who employed MACI for similar patients. These results encompass both pain relief and joint functionality [49,50]. As HD-ACI places a strong emphasis on cell density, it holds the potential to enhance tissue quality, although the availability of direct comparative studies between HD-ACI and other techniques is limited. Consequently, drawing conclusive assertions regarding its superiority proves to be a challenge. While HD-ACI prioritizes high cell density, other methods such as ACI with periosteal flap and MACI also involve cell introduction, albeit with differing focal points. Both MACI and HD-ACI employ a scaffold, which could potentially offer superior mechanical support compared to the periosteal flap utilized in ACI. This choice has the potential to significantly impact stability and long-term outcomes. It is worth noting that HD-ACI, ACI with periosteal flap, and MACI share a degree of complexity due to the intricacies of cell culturing and implantation procedures. This complexity may inherently influence both surgical results and patient recovery.

**Figure 8:**
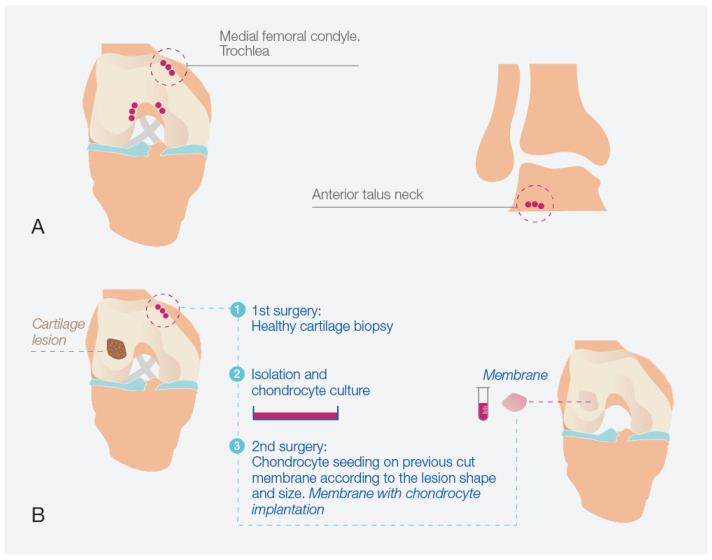
High-density autologous chondrocyte implantation (HD-ACI) process. (**A**) Identification of non-weight bearing regions in the knee or ankle for biopsy extraction. (**B**) Schematic representation of the HD-ACI approach applied to the knee, comprising two sequential surgical stages: initial biopsy collection, chondrocyte isolation, and culture in the first surgery, followed by the second surgery involving precise placement of cells onto a pre-shaped membrane tailored to fit the lesion’s dimensions and configuration, achieving a cell density of 5 million cells per cm^2^.

Figure 8 illustrates the schematic representation of the HD-ACI procedure. The process commences with an initial surgical step involving the extraction of a biopsy from a non-weight-bearing area of the joint that is in a healthy condition. These areas typically include the medial femoral condyle or trochlea for the knee and the anterior talus neck for the ankle (Figure 8A). The acquired biopsy is then transported to a GMP-certified laboratory for processing. Here, chondrocytes are isolated and cultured until a sufficient cell count is achieved based on the dimensions of the cartilage lesion. This cultivation period typically spans 4 to 6 weeks. The subsequent surgical step is represented in Figure 8B, encompassing the implantation of the cultured chondrocytes. During this procedure, the cartilage lesion is exposed and meticulously debrided to ensure healthy edges. Subsequently, a porcine type I/III collagen membrane is tailored to fit the shape and size of the cartilage lesion. This membrane comprises a rough surface and an impermeable smooth side, preventing chondrocyte escape. The cultured chondrocytes are carefully placed onto the rough surface of the membrane at a density of 5 million cells per cm^2^ of cartilage lesion. After a 12-min incubation period (allowing for chondrocyte absorption by the collagen carrier), the membrane is positioned over the cartilage defect and carefully sutured to the surrounding healthy cartilage. The attachment is further secured using fibrin glue. The stability of the membrane is verified through flexion–extension movements. Patients undergoing this procedure for the knee, ankle, or hip adhere to specific post-operative protocols tailored to the respective joint. These protocols encompass a gradual mobilization regimen starting with non-weightbearing, followed by incremental weightbearing with crutches, initiation of physical therapy sessions, and progressive limb mobilization.

One of the limitations we encountered in our clinical studies involving HD-ACI is the relatively small number of patients who participated, coupled with the short follow-up period. Focal cartilage lesions in young individuals with high-performance joints, suitable candidates for HD-ACI, are not widespread, which initially led to a limited patient pool in our studies. However, as time has progressed, the enrollment of patients in our studies has been steadily increasing. Ongoing, long-term assessments are being diligently conducted to thoroughly assess the safety and effectiveness of the procedure as patients are continuously monitored over an extended timeframe. This ongoing monitoring will yield valuable insights into the long-term outcomes of HD-ACI.

Another limitation of the HD-ACI technique arises from its two-step nature. To address this concern, we are exploring a potential solution: the development of an allogeneic therapy that capitalizes on the unique properties of hyaline cartilage, which lacks blood and lymphatic vessels. Our current research is concentrated on employing allogeneic chondrocytes for treating cartilage defects. We are actively planning to initiate a clinical trial in the near future to evaluate the safety and efficacy of this approach. One of the initial problems that arises in culturing cells for an allogeneic therapy is to manufacture chondrocytes under GMP conditions to allow cell proliferation obtaining a large-scale culture retaining the cells their properties. A first attempt at culturing cells in a hollow fiber bioreactor and with GMP compliance has been recently published [51].

HD-ACI represents a method for repairing cartilage with the aim of achieving resilient hyaline cartilage restoration, based on monolayer-cultured chondrocytes. Methods for enhancing cartilage organization and mechanical properties encompass various strategies, including mechanical stimulation, cultivation in hypoxic environments, and the application of large molecules such as growth factors. Additionally, cutting-edge technologies such as bioreactors, biosensors, and 3D bioprinting are under active investigation. All of these tissue engineering approaches will contribute to the advancement and fine-tuning of therapeutic development [52,53,54]. They could have some peculiar characteristics that provide them to some differences to the traditional two-dimensional cultures: they could mimic in vivo conditions, providing a scaffold for cells to grow within, allowing them to interact with neighboring cells and extracellular matrix components in a manner similar to in vivo conditions and establishing more natural cell-to-cell interactions.

Given that these strategies are primarily tailored for the treatment of localized cartilage injuries, the future of regenerative medicine holds the potential to expand its scope to address degenerative cartilage conditions like osteoarthritis. In such conditions, cellular senescence plays a pivotal role in the breakdown of the extracellular matrix and tissue deterioration [55,56]. Cellular senescence is a complex process that halts cell proliferation. Thus, if we can uncover means of reversing senescence, there is potential to rejuvenate the population of cells affected by osteoarthritis and impede the progression of the disease. Notably, Katoh et al. [57] have introduced a method that, within a 3D chondrocyte culture employing a polymer scaffold, appears to have the capacity to reverse senescence without inducing alterations in the cell genome.

As we peer into the next 5–10 years, we discern several captivating avenues that this field could traverse. The realm of advanced techniques and materials beckons us forward. We should anticipate research and innovation to unveil more refined methodologies and biomaterials for precise cell delivery-think innovative scaffolds, growth factors, and inventive mechanisms for introducing cells. All of these elements are harmonized to enhance the efficiency of the implantation process. Simultaneously, the frontier of regenerative medicine continues its march toward personalization. In the coming years, the pursuit of tailoring HD-ACI treatments could intensify. This personalized approach could be fine-tuned for individual patients, considering factors like age, severity of condition, and distinct joint characteristics. This tailored strategy holds the potential to yield more resounding successes, perhaps even expediting the path to recovery.

The relentless quest for enhanced long-term outcomes persists. Unwavering efforts in research and clinical trials may refine the long-term success rates of HD-ACI. Within the realm of medicine, practitioners will trace patient progress over years, illuminating deeper insights into the treatment’s endurance and true efficacy.

The trajectory of surgical techniques is also evolving. We should anticipate advancements in surgical expertise that unlock less invasive avenues for administering high density chondrocyte implants. We can envision reduced patient discomfort, swifter recoveries, and a broader reach, ensuring a more diverse range of individuals who can benefit from this innovation.

Lastly, we contemplate the landscape of regulatory approvals. Over the upcoming 5–10 years, the stamp of approval from regulatory bodies could lend credibility to novel HD-ACI methods, including allogeneic therapies. This endorsement could propel these methods into the realm of available treatments, fostering wider adoption and accumulating a wealth of real-world data.

## Figures and Tables

**Table 2:** Type II collagen and aggrecan expression in membrane pieces and supernatants after incubation with the different cell densities assayed. In this experiment, 1 cm^2^ collagen membrane pieces were incubated with different chondrocyte amount (from 1 million to 10 million cells). After incubation, supernatants were removed and tested for both genes’ expression.

Cell Density	Type II Collagen		Aggrecan	
	Supernatant	Membrane	Supernatant	Membrane
1 million/cm^2^	Negative	Positive	Negative	Positive
2 million/cm^2^	Negative	Positive	Negative	Positive
3 million/cm^2^	Negative	Positive	Negative	Positive
4 million/cm^2^	Negative	Positive	Negative	Positive
5 million/cm^2^	Negative	Positive	Negative	Positive
6 million/cm^2^	Positive	Positive	Positive	Positive
7 million/cm^2^	Positive	Positive	Positive	Positive
8 million/cm^2^	Positive	Positive	Positive	Positive
9 million/cm^2^	Positive	Positive	Positive	Positive
10 million/cm^2^	Positive	Positive	Positive	Positive

## Data Availability

No new data were created or analyzed in this study. Data sharing is not applicable to this article.
